# Archaeal tRNA-Splicing Endonuclease as an Effector for RNA Recombination and Novel Trans-Splicing Pathways in Eukaryotes

**DOI:** 10.3390/jof7121069

**Published:** 2021-12-12

**Authors:** Giuseppe D. Tocchini-Valentini, Glauco P. Tocchini-Valentini

**Affiliations:** 1 Istituto di Biochimica e Biologia Cellulare, Campus Internazionale “A. Buzzati-Traverso”, Dipartimento Scienze Biomediche, Consiglio Nazionale delle Ricerche, via Ramarini 32, 00015 Monterotondo, Rome, Italy; glaucotocchini@gmail.com; 2 Dipartimento Scienze Biomediche, European Mouse Mutant Archive (EMMA), INFRAFRONTIER-IMPC, Monterotondo Mouse Clinic, Campus Internazionale “A. Buzzati-Traverso”, Consiglio Nazionale delle Ricerche, via Ramarini 32, 00015 Monterotondo, Rome, Italy

**Keywords:** tRNA-splicing endonuclease, RNA processing, RNA recombination

## Abstract

We have characterized a homodimeric tRNA endonuclease from the euryarchaeota *Ferroplasma acidarmanus* (FERAC), a facultative anaerobe which can grow at temperatures ranging from 35 to 42 °C. This enzyme, contrary to the eukaryal tRNA endonucleases and the homotetrameric *Methanocaldococcus jannaschii* (METJA) homologs, is able to cleave minimal BHB (bulge–helix–bulge) substrates at 30 °C. The expression of this enzyme in *Schizosaccharomyces pombe* (SCHPO) enables the use of its properties as effectors by inserting BHB motif introns into hairpin loops normally seen in mRNA transcripts. In addition, the FERAC endonuclease can create proteins with new functionalities through the recombination of protein domains.

## 1. Introduction

Nature employs various mechanisms to remove introns from mRNA, tRNA, and rRNA. In bacteriophages, bacteria, chloroplasts and mitochondria, self-splicing group I and II introns are found [1,2,3]. In the eukaryotic nucleus, mRNA introns are removed by the spliceosome [4,5], and tRNA introns are removed by the combined action of the tRNA endonuclease and the tRNA ligase [6,7,8,9].

In Archaea, unlike bacteria and eukaryotes, all introns, whether in pre-tRNA or elsewhere, use an intron excision mechanism based solely on the tRNA-splicing endonuclease and tRNA ligase [10,11,12,13]. This property is due to the ability of the archaeal tRNA endonuclease to recognize two distinct motifs: the first contains a 2 or 3 nt bulge separated by a 4 bp helix (BHB motif), and the second contains a 3 nt bulge and an internal loop separated by a 4 bp helix (BHL-like motif), regardless of the presence of a tRNA mature domain [14,15]. These motifs can be found in pre-tRNA in the anticodon loop, as well as in non-canonical positions such as the D-arm and T-arm loops [16,17,18], but they can also be found in primary transcripts of the rRNA operon, which code for 16S and 5S rRNA [11,12,19]. 

These two types of substrates are processed by four different types of tRNA endonuclease: a homotetramer in some Euryarchaea, a homodimer in other Euryarchaea, and a heteroteramer in Crenoarchaea and Nanoarchaea [17,20,21,22,23]. Based on the phylogenetic distribution of the motifs at exon–intron junctions and endonuclease architectures, it has been concluded that all the different forms of the enzyme can cleave the canonical BHB and that only the homodimeric and heterotetrameric forms can cleave the BHL-like motif [17,18].

The current study aims to use specific RNA products produced by the expression of an exogenous archaeal tRNA endonuclease to target genes implicated in genetic and multifactorial pathogenesis in eukaryotes. Our findings show that it is unique in that it does not involve altering the unwanted gene’s DNA sequence, transcriptional regulation, or mRNA maturation. Moreover, the FERAC endonuclease is a promising method for synthetic biology and might be used to build chimera combinations of diverse transcripts. 

## 2. Materials and Methods

### 2.1. tRNA-Splicing Endonuclease Purification

The enzymes, a homodimer from METJA, a heterotetramer from FERAC, and a heterotetramer from SCHPO, were purified as previously described [24]. Molecular concentration of the purified proteins was determined by measuring the protein concentration using absorbance at 280 nm and the calculated extinction coefficients.

### 2.2. RNA Synthesis In Vitro and RNA Cleavage Reactions

Synthesized DNA templates were used in T7 RNA polymerase transcription reactions carried out with the Ambion T7-Megashortscript kit. [α-32P]UTP (800 Ci/mmol; Perkin–Elmer, Waltham, MA, USA) was included in the reaction to label the transcription products [12,25,26,27,28]. Transcripts of the correct size were purified by electrophoresis on a 10% (*wt*/*vol*) denaturing polyacrylamide gel, followed by elution, phenol extraction, and ethanol precipitation. The same conditions were used for all of the reactions: 25 mM Tris-HCl (pH 7.5), 5 mM MgCl_2_, 100 mM NaCl, and 10% (*vol*/*vol*) glycerol, 20 fmol of substrate, and equimolar amounts of tRNA endonuclease (2 μM). The reactions were incubated at 30 °C or 65 °C for 8 min. Aliquots were pooled at 2 min intervals, the reaction was stopped by phenol extraction and the ethanol precipitated [12]. The products were separated on 10% (*wt*/*vol*) denaturing polyacrylamide gels and analyzed on a Molecular Dynamics model Storm 860 PhosphorImager using ImageQuant software, version 4. Local average background was corrected, and the fraction cleaved was calculated by the ratio of cleaved product to the sum of the cleaved product plus uncleaved substrate [25].

### 2.3. SCHPO Strains and Media

The fission yeast strain used in the present study was IHSP365 (*h^−^* *leu1-32 ura4-D18*). Cells were grown in YE (0.5% yeast extract and 3% glucose) plus appropriate supplements. Yeast cells carrying a plasmid were grown in EMM (Edinburgh minimal medium, Formedium, Norfolk, UK) plus appropriate supplements [29]. Standard protocols for genetic manipulation of fission yeast were used as described in [29].

### 2.4. Vector Construction

All vectors were constructed following PCR amplification of desired genes and their insertion between the NdeI and BamHI sites of either plasmid pREP41-MHN or pREP42-MHN [30]. The clones were verified by sequencing. The genes are under the control of a thiamine-regulated nmt promoter; the vector pREP41 contains the auxotrophic LEU2 marker, while pREP42 contains the URA4 marker.

### 2.5. Preparation of Cell Extracts and Western Blots

Cell extracts were prepared according to [30], resolved by SDS-PAGE, transferred onto immobilon PVDF transfer membranes and then analyzed by Western blot, using a primary antibody specific for the *Myc*-tag. Horseradish peroxidase-conjugated secondary antibodies were detected using SuperSignal ULTRA Chemiluminescent Substrate (Pierce, Holmed, NJ, USA).

### 2.6. Motif Search for a Hairpin Loop

Using a descriptor for a hairpin consisting of a six-base pair helix and a seven-nucleotide loop motif, the RNABOB program [31] was used to search the mRNA sequence of galactosidase. This secondary structure was derived from the anticodon arm of tRNAs.

### 2.7. β-Galactosidase Assay

The ability of each tRNA endonuclease to process a BHB-lacZ, restoring the β-galactosidase activity, was determined. To measure the activity quantitatively, the cells were grown in liquid cultures to mid-log phase at 30 °C in EMM containing appropriate supplement and 4 mM thiamine. The cells were then washed with thiamine-free medium and re-inoculated into EMM in the absence of thiamine and grown to mid-log phase. The assay of the β-galactosidase activity was performed as described in [32,33] using o-nitrophenyl *β*-D-galactopyranoside (ONPG, Sigma, Saint Louis, MO, USA) as substrate.

### 2.8. RNA Extraction and RT PCR

Cells were grown in selective medium and harvested in mid-log phase, resuspended in 20 mM Tris-HCl pH 7.5, 100 mM NaCl, 1 mM EDTA, and lysed by vortexing with glass beads in the presence of phenol. After centrifugation, ethanol was used to precipitate RNA from the aqueous phase. A 5 mg aliquot of the extracted RNA was reverse transcribed (RT) with a primer specific for the donor sequence (5′CGCGGATCCTCAATTAGGGGCAGGGCATGCTC). Subsequently, the DNA was used as a template for PCR amplification by using an upstream primer specific for the acceptor sequence and the oligomer previously used for the RT as a downstream primer. The RNA was reverse transcribed using SuperScript III reverse transcriptase (Invitrogen) at 55 °C, followed by PCR amplification with Taq DNA polymerase using a forward primer (5′CTCACTTACGGGCCATATGTGGTCTTCCTTTCAGC) and as a reverse primer the primer used for the RT reaction. Products were analyzed by electrophoresis on 2% agarose gels containing ethidium bromide in TBE buffer (80 mM Tris base, 90 mM Boric acid, 1 mM EDTA).

## 3. Results

### 3.1. The FERAC Homolog of the tRNA-Splicing Endonuclease

The kingdom of Archaea is characterized by microorganisms capable of living in harsh environments, such as high temperatures, organic solvents, high salt concentrations, or acidic or alkaline conditions. We sought an enzyme with features more compatible with the temperature requirements typical of eukaryotes. The euryarchaeota FERAC is a facultative anaerobe, whose optimal growth temperature is between 35 and 42 °C [34]. In a previous study [17], we identified, using the sequence of the METJA (Q58819) enzyme as a probe in BLASTP (Basic Local Alignment Search Tool Program) searches, a gene coding for the protein S0AS78 in the genome of FERAC. The size of the protein, 288 residues, and our phylogenetic studies suggested that we were dealing with a homodimeric enzyme [17]. The gene was cloned in pET28 to obtain a construct that coded for a protein with a modified N terminus presenting a His-6 tag. The tagged enzyme was purified by affinity chromatography followed by gel filtration chromatography. It eluted as a single peak corresponding to the size of the predicted protein, as described in the Section 2.

### 3.2. The FERAC tRNA Endonuclease Can Cleave the Mini BHB Motif Substrate at 30 °C In Vitro

**Figure 1:**
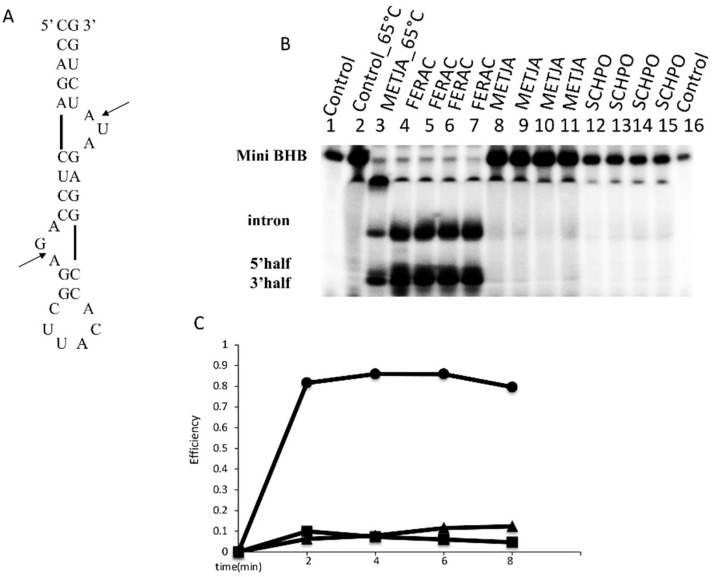
RNA cleavage assay. (**A**) Mini BHB substrate for the in vitro cleavage assay. Arrows indicate the splicing sites. (**B**) The mini BHB substrate was incubated with three different enzymes, in the reaction conditions as reported in the Section 2. The cleavage products were analyzed by electrophoresis on 10% polyacrylamide gel containing 29:1 monomer to bis and 8 M urea, followed by autoradiography. The identification of the reaction products is indicated. Lane 1–2 contain the control (C, no enzyme added) incubated at 30 °C and 65 °C. Lanes 4–7, 8–11, and 12–15 show the products after incubation with the endonucleases from FERAC, METJA, and SCHPO, respectively. Lane 3 shows the products following incubation at 65 °C in the presence of the METJA enzyme. Lane 16 contains purified T7 transcribed substrate not being incubated. (**C**) Splicing plateau levels showing the functionality of each of the three enzymes at 30 °C. All reactions were performed at 1 mM RNA and 2 mM enzyme under the conditions reported in the Section 2. Δ, SCHPO; □, METJA; O, FERAC.

We compared the cleavage activities, at 30 °C and 65 °C, of three purified enzymes: the FERAC tRNA endonuclease, the homotetrameric METJA enzyme and the eukaryal heterotetrameric tRNA endonuclease of SCHPO. Equimolar amounts of enzyme (2 μM) were incubated in the presence of the mini BHB, uniformly labeled with ^32^P (Figure 1A) substrate (20 fmol) for 8 min. Aliquots were withdrawn at 2 min intervals, the reaction was stopped and products were resolved on a denaturing gel, as described in the Section 2. Figure 1B shows that in the absence of enzyme, no cleavage occurred at either temperature (lanes 1–2), as expected. However, the FERAC enzyme was able to correctly cleave the mini BHB substrate at 30 °C (lanes 4–7), while the METJA and SCHPO enzymes did not (lanes 8–11 and 12–15, respectively). When the METJA enzyme reaction was run at 65 °C (lane3) the substrate was cleaved, showing a striking temperature dependence. Interestingly, the SCHPO enzyme, which should be active at 30 °C, performed extremely poorly.

In the absence of kinetic determinations to compare the efficiency of the tested enzymes, we quantified the reaction products as specified in the Section 2. Figure 1C shows that the FERAC enzyme rapidly cleaves all of the substrate. This indicates that the RNA substrate is not misfolded and that it can be processed to completion. The SCHPO and METJA enzymes performed poorly, plateauing slightly above background.

### 3.3. In Vivo Expression of FERAC tRNA Endonucleases in SCHPO

Endonuclease efficiency was assessed in SCHPO because of the organism’s endogenous heterotetrameric subunit genes and the possibility to clone, express, and compare the efficiency of the archaeal enzymes’ homotetrameric and homodimeric versions using only a single vector.

**Figure 2:**
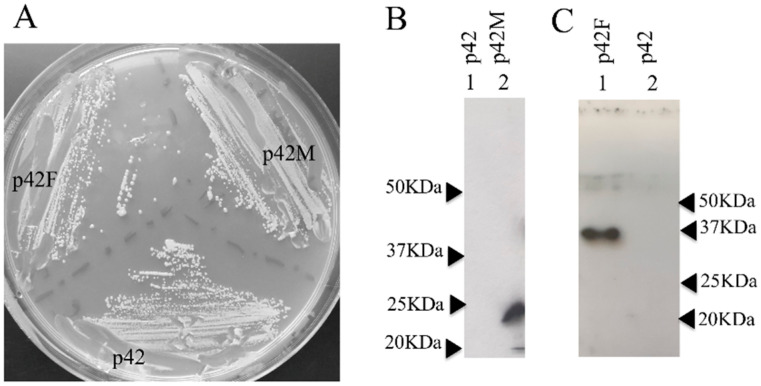
Analysis of growth and expression of archaeal tRNA endonuclease in SCHPO. (**A**) SCHPO expressing either the FERAC endonuclease (p42F) or the METJA enzyme (p42M), or no enzyme (p42) growing on EMM in absence of thiamine at 30 °C. (**B**) Detection by Western blot using an anti-Myc antibody of Myc-tagged METJA (lane2) tRNA endonuclease and the control (lane1) in extracts from cells grown in the absence of thiamine. (**C**) Detection by Western blot using an anti-Myc antibody of Myc-tagged FERAC (lane 1) tRNA endonuclease, and the control (lane 2) in extracts from cells grown in the absence of thiamine.

As a first step, the genes coding for the METJA and FERAC enzymes were cloned into pREP42-HMN under the control of a medium-strength promoter and a transcriptional termination element of the thiamine-repressible gene nmt1 [30,35,36]. This vector enables the episomal expression of a tRNA endonuclease fused to tag sequences. The expressed protein presents an N-terminal tag comprising two copies of the Myc epitope and six histidine residues [30]. The SCHPO strain IHSP365 with genotype *h− leul.32 ura4.d18* was used to determine if the expression of the exogenous protein could be toxic to SCHPO. The toxicity was tested by streaking the transformed cells on plates with different concentrations of thiamine [37]. Figure 2A shows that expressing the enzyme from FERAC (p42F), METJA (p42M), and the empty vector (p42) in the absence of thiamine, therefore under maximal inducing conditions, is not deleterious to SCHPO growth. To investigate the expression levels of the enzymes, Western blot analysis of whole cell extracts was carried out using an antibody specific for the Myc-tag [30] (Figure 2B,C). No strongly immunoreactive proteins were detected in lane 1B and 2C (the vector control); moreover, the antibody gave a negligible background. On the contrary, in lanes 2B and 1C, single bands of the predicted size were detected, showing that the expression of the fusion proteins was successful.

### 3.4. The FERAC tRNA Endonuclease Mediates RNA Trans-Recombination to Form Chimeric mRNA In Vivo

**Figure 3:**
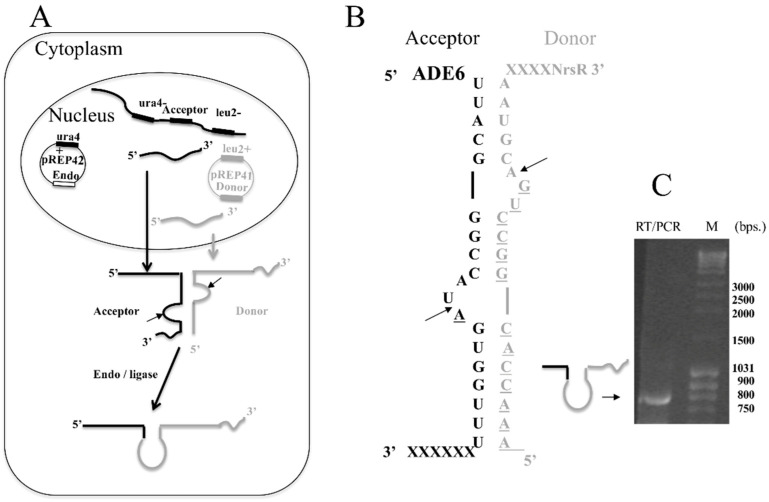
RNA recombination. (**A**) Schematic view of the gene targeting strategy. (**B**) BHB motif necessary for the tRNA endonuclease-dependent recombinatorial event. The sequence belonging to the endogenous acceptor gene is in black; the sequence of the donor is in gray. (**C**) RT-PCR analysis by agarose gel electrophoresis of the recombination product.

Figure 3A summarizes the technique used in vivo. Two transcripts, one expressed episomally and designated as the donor, and one encoded in the genome and designated as the acceptor, combine to generate a BHB that serves as the substrate for the exogenous FERAC tRNA endonuclease. The SCHPO leucine and uracil autotrophic strain IHSP365 was transformed with two compatible vectors, pREP42, which contains the tRNA endonuclease gene, and pREP41, which contains a sequence whose transcript (donor) cannot code for a full protein unless it forms a BHB motif with an endogenous mRNA transcript (acceptor).

As an acceptor we used the endogenous mRNA transcript of the gene that codes for the enzyme N-succinyl-5-aminoimidazole-4-carboxamide ribotide synthetase (ADE6) [38].

**Figure 4:**
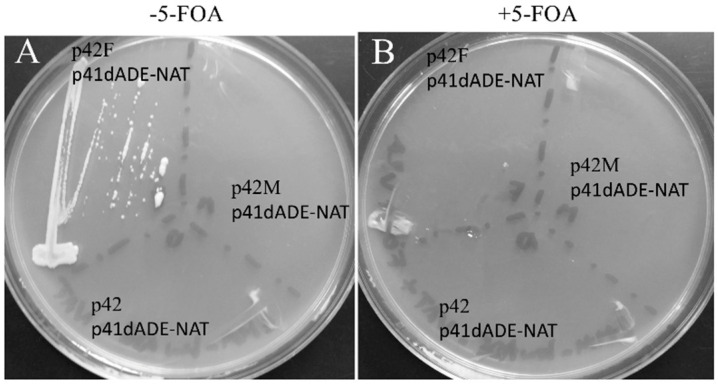
Sensitivity and resistance. (**A**) SCHPO cells co-transformed with pREP41-dADE-NAT and either a pREP42 vector empty or expressing FERAC or METJA tRNA endonucleases were streaked on EMM containing plates containing 100 µg/mL of nourseothricin (NAT) and adenine in absence of thiamine at 30 °C for three days. (**B**) The same strains were streaked on the same medium containing 5-FOA.

Yeast harboring the mutation of this gene can survive only if adenine is present in the growth medium. The donor is a gene that codes for *Streptomyces noursei* nat1, encoding an N-acetyltransferase (NrsR) that monoacetylates nourseothricin (NAT), an inhibitor of ribosomal protein synthesis that induces miscoding during translation in a wide range of prokaryotic and eukaryotic organisms [39,40,41]. This gene, cloned into pREP41-HMN, presents an upstream sequence designed to form a BHB structure upon interaction with the ADE6 mRNA (Figure 3B). The NrsR ORF is out of frame and can be translated only if the BHB structure is formed, and is cleaved by a tRNA endonuclease. To examine the splicing product, we analyzed the final RNA product by RT-PCR. The product of the reaction was analyzed by electrophoresis on an agarose gel, Figure 3C shows a band that in size corresponds to the recombinant RNA. The translational product resulting from the recombinatorial event between ADE6 and NrSR mRNA should be a chimeric protein that presents the N-terminus of ADE6 and, at its C-terminus, the NrsR protein that confers resistance to NAT. SCHPO strains transformed with pREP41dADE_NAT and either with pREP42-HMN or pREP42-METJA (p42M) or pREP42-FERAC (p42F) were streaked on EMM plates containing NAT and adenine, in the absence of thiamine. Only the strain carrying the plasmid p42F (Figure 4A) was resistant to the antibiotic, as a consequence of the action of the FERAC enzyme. The strains were also streaked on the same medium but this time containing 5-fluoroorotic acid (5-FOA) and uracil at low concentration. Because the plasmid pREP42 contains a ura4^+^ selectable marker encoding orotidine 5’-phosphate decarboxylase that converts 5-FOA into the toxic compound 5-fluorouracil, causing cell death, cells harboring this vector cannot grow unless they lose the plasmid. Figure 4B shows that the strain expressing the FERAC endonuclease cloned into pREP42 was now sensitive to the 5-FOA.

### 3.5. Intron Regulation of Gene Expression

**Figure 5:**
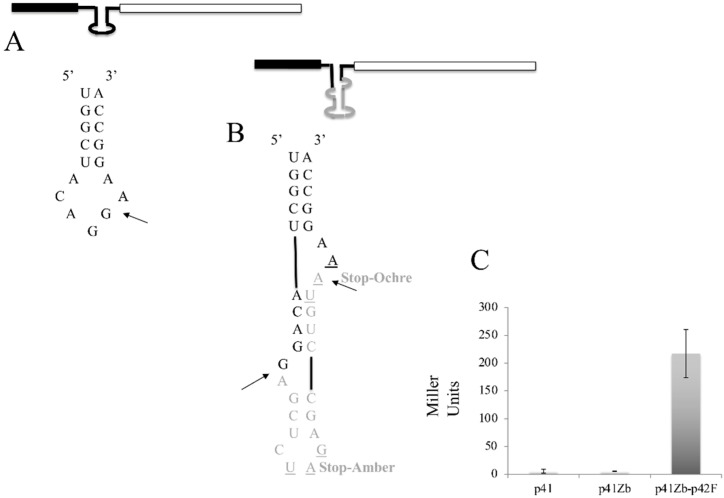
β-galactosidase activity of the gene construct pREP41-Zb. (**A**) Internal hairpin loop. (**B**) The transcribed gene presents an intron so that it cannot be translated. Only the correct removal of the intron generates a transcript that can be successfully translated. The sequence belonging to the gene is in black, the intron sequence is in gray. (**C**) Quantitative β-gal assay of the SCHPO strain carrying only the pREP41-Zb (p41Zb) or the control vector alone (p41) or co-transformed with pREP42-FERAC (p41Zb-p42F).

To produce a substrate that is cleaved only by the FERAC enzyme, we decided to use an in vivo approach based on the β-galactosidase (lacZ) assay [32,33]. As a first step, the mRNA sequence of lacZ was searched, using the RNABOB program [31] with a descriptor (Section 2), to find a hairpin presenting a six-base pair helix and a seven-nucleotide loop (Figure 5A). This step is necessary for two reasons: first, the hairpin resembles the anticodon arm structure of a mature tRNA, and therefore should facilitate the action of the endogenous tRNA ligase following the removal of the intron; second, it does not require the introduction of an exogenous hairpin that could otherwise impair either the transcription or the translation of the mRNA encoded by the target gene. We decided to insert into the hairpin of the β-galactosidase gene an intron capable of folding into a BHB structure. This BHB motif presents a two nucleotides bulge at the 5′ splicing site and a three nucleotides bulge at the 3′ separated by a 4-bp helix (Figure 5B). A β-galactosidase gene presenting such an intron was cloned into pREP41-HMN (p41Zb). The presence of the intron results in a transcript that is out of frame and cannot be translated into β-galactosidase. The strain IHSP365 was transformed with the vector p41Zb, in the absence or the presence of p42F, and as a control the same strain was transformed with the p41 vector alone. Transformant colonies were selected on EMM in the case of the strain carrying p42F and p41Zb, while cells carrying only p41Zb or p41 were selected on EMM containing uracil. To accurately monitor the regulation of the expression of the genes under test, we used a β-galactosidase in vitro assay to provide a sensitive and quantitative assessment of β-galactosidase activity by measuring the hydrolysis of the Ortho-nitrophenyl-β-D-galactopyranoside (ONPG) substrate into galactose and the chromogenic compound orthro nitrophenol.

In Figure 5C, we show that Miller units calculated for both controls p41 and p41Zb were less than 5, implying the absence of β-galactosidase expression, while the strain carrying both p42F and p41Zb plasmids yielded 216 Miller units of β-galactosidase.

## 4. Discussion

Previous studies of the activity of tRNA endonucleases in eukaryotic cells were published. A series of studies looked at the expression in human cells of the homotetrameric tRNA endonuclease from a METJA [42,43,44], a thermophilic methanogenic archaean growing at temperatures between 48 °C and 94 °C. The optimal temperature of the enzyme limited the efficiency of the reaction. In other studies, the endogenous tRNA endonuclease and ligase from *Saccharomyces cerevisiae* and in mouse cells were employed to trans-splice two different transcripts together in a chimera by the forming of a pretRNA structure [45,46]. Regrettably, due to the requirement of a pretRNA, the tRNA endonuclease cannot target genomic endogenous mRNA, and its expression cannot be controlled to calibrate efficiency.

A strategy with significant advantages must: first, act just at the mRNA level, involving no genomic alterations; and second, be regulated by modulating the expression of the tRNA endonuclease, which works at temperatures ranging from 30 to 37 °C.

We show in this work that the homodimeric archaeal FERAC tRNA endonuclease is a suitable choice for developing into a new synthetic biology tool for trans-splicing to create novel chimera combinations of various transcripts since it exhibits all of the aforementioned features. The FERAC tRNA endonuclease can be used to investigate protein function by creating mRNA chimeras via trans-splicing joining after the formation of a BHB structure. The formation of this structure and high specificity of the enzyme, when coupled with an optimal screening of sequence homologies in the genome, considerably decreases the possibility of nonspecific trans-splicing to other endogenous mRNA. Not only can mutations be inserted at specific locations within a protein, but protein domains can also be combined to produce new functions. The gain of NAT resistance as a result of chimeric mRNA translation reveals how efficiently and properly the tRNA endonuclease processes the substrates, as shown by the reverse transcription polymerase chain reaction product.

The FERAC endonuclease also provides a cis-splicing mechanism that recognizes a BHB motif as the major determinant for its recruitment and is virtually sequence independent.

The efficacy of this regulatory mechanism at the level of transcription, processing, and translation is revealed by restoring the β-galactosidase activity following the precise ablation of the intron.

In higher eukaryotes, this potential could be realized by expressing the endonuclease with tissue-specific promoters in certain cell types and tissues.

Similarly, this approach may be used to tag a target protein intercating in a complex without affecting its natural expression, lowering the likelihood of side effects from overexpression.

The fact that the activity of FERAC endonuclease in vivo is dependent on its interaction with two biological components of the splicing reaction, the target mRNA and an endogenous RNA ligase, suggests a possible framework for future research to improve efficiency.

Our next goal will be to extend these studies in animal models.
